# The trace amine-associated receptor 1 modulates methamphetamine's neurochemical and behavioral effects

**DOI:** 10.3389/fnins.2015.00039

**Published:** 2015-02-13

**Authors:** Rachel Cotter, Yue Pei, Liudmila Mus, Anja Harmeier, Raul R. Gainetdinov, Marius C. Hoener, Juan J. Canales

**Affiliations:** ^1^Department of Psychology, University of CanterburyChristchurch, New Zealand; ^2^Behavioural Neuroscience, School of Psychology, University of LeicesterLeicester, UK; ^3^Department of Neuroscience and Brain Technologies, Istituto Italiano di TecnologiaGenoa, Italy; ^4^Neuroscience Research, Pharmaceuticals Division, F. Hoffmann-La Roche Ltd.Basel, Switzerland; ^5^Skolkovo Institute of Science and TechnologySkolkovo, Moscow, Russia; ^6^Faculty of Biology, St. Petersburg State UniversitySt. Petersburg, Russia

**Keywords:** trace amine-associated receptor, methamphetamine, sensitization, self-administration, synaptosomes, microdialysis

## Abstract

The newly discovered trace amine-associated receptor 1 (TAAR1) has the ability to regulate both dopamine function and psychostimulant action. Here, we tested in rats the ability of RO5203648, a selective TAAR1 partial agonist, to modulate the physiological and behavioral effects of methamphetamine (METH). In experiment 1, RO5203468 dose- and time-dependently altered METH-induced locomotor activity, manifested as an early attenuation followed by a late potentiation of METH's stimulating effects. In experiment 2, rats received a 14-day treatment regimen during which RO5203648 was co-administered with METH. RO5203648 dose-dependently attenuated METH-stimulated hyperactivity, with the effects becoming more apparent as the treatments progressed. After chronic exposure and 3-day withdrawal, rats were tested for locomotor sensitization. RO5203648 administration during the sensitizing phase prevented the development of METH sensitization. However, RO5203648, at the high dose, cross-sensitized with METH. In experiment 3, RO5203648 dose-dependently blocked METH self-administration without affecting operant responding maintained by sucrose, and exhibited lack of reinforcing efficacy when tested as a METH's substitute. Neurochemical data showed that RO5203648 did not affect METH-mediated DA efflux and uptake inhibition in striatal synaptosomes. *In vivo*, however, RO5203648 was able to transiently inhibit METH-induced accumulation of extracellular DA levels in the nucleus accumbens. Taken together, these data highlight the significant potential of TAAR1 to modulate METH's neurochemical and behavioral effects.

## Introduction

Methamphetamine (METH) is a widely abused and highly addictive psychostimulant drug. In the brain, METH elevates extracellular dopamine (DA) levels, an effect that is thought to underlie its potent motor and psychoactive actions (Fleckenstein et al., 2007). METH competes with DA for reuptake and causes reverse transport through the DA transporter (DAT) (Elliott and Beveridge, 2005; Goodwin et al., 2009). In addition, methamphetamine depletes monoamines from vesicular storage through the vesicular monoamine transporter-2 (VMAT2), resulting in increased DA in the cytoplasm available for reverse transport by the DAT (Sulzer et al., 2005; Fleckenstein et al., 2009). Apart from its direct interaction with the DA system, METH is also a potent agonist at the trace amine-associated receptor 1 (TAAR1) (Bunzow et al., 2001; Reese et al., 2007). TAAR1 belongs to a family of G-protein coupled receptors that is activated by trace amines (TAs), a group of endogenous amines often referred to as “endogenous amphetamines” due to their ability to increase monoamine release via displacing monoamines from synaptic vesicles and reversing monoamine transporters on the plasma membrane (Berry, 2004). TAs have been implicated in brain reward and in the reinforcing properties of psychostimulants (Gilbert and Cooper, 1983; Shannon and Thompson, 1984), albeit their independent role as neurotransmitters was not recognized until TAAR1 was discovered and characterized (Borowsky et al., 2001; Bunzow et al., 2001). TAAR1 shares overlapping distribution in the brain with mesolimbic DA pathways (Borowsky et al., 2001; Lindemann et al., 2008), is co-localized with the DAT in a subset of DA neurons (Xie and Miller, 2007), and interacts with both the DAT and the D2 DA autoreceptor (D2R) to modulate DA transmission (Xie et al., 2007, 2008; Espinoza et al., 2011). These observations suggest that TAAR1 regulates DA activity and has the potential to serve as a pharmacological target to modulate DA dysregulation that results from chronic stimulant exposure.

Early evidence supporting the potential of TAAR1 to control DA function comes from studies on Taar1 knock-out mice which showed an elevated spontaneous firing rate of DA neurons in the ventral tegmental area (Lindemann et al., 2008) and increased DA release in the NAc (Leo et al., 2014), suggesting that TAAR1 is constitutively active or tonically activated by ambient levels of amines, including TAs, to negatively regulate DA activity. Those mutant mice also displayed enhanced sensitivity to psychostimulant-induced locomotor activity, conditioned place preference (Achat-Mendes et al., 2012) and striatal DA release (Wolinsky et al., 2007; Lindemann et al., 2008), supporting the role of TAAR1 in psychostimulant action. The recent development of several selective TAAR1 agonists has enabled a more direct assessment of TAAR1 functionality. While the TAAR1 full agonists, RO5166017 (Revel et al., 2011) and RO5256390 (Revel et al., 2012a), were able to inhibit the firing rate of dopaminergic neurons in the ventral tegmental area, the TAAR1 partial agonists, RO5203648 (Revel et al., 2012b) and RO5263397 (Revel et al., 2012a), and the antagonist, EPPTB (Bradaia et al., 2009), increased their firing frequency, demonstrating a strong modulation of DA activity by TAAR1 activation.

More recent studies have focused on examining the therapeutic-like effects of TAAR1 agonists on cocaine addiction. It was found that TAAR1 activation suppressed cocaine-induced locomotor hyperactivity (Revel et al., 2012b), behavioral sensitization and conditioned place preference (Thorn et al., 2014), and reduced cocaine self-administration (Revel et al., 2012b; Thorn et al., 2014), suggesting a blockade of both acute psychostimulating and long-term psychomotor sensitization effect of cocaine as well as cocaine's reinforcing property through pharmacological activation of TAAR1. Moreover, the two partial agonists, RO5203648 (Pei et al., 2014) and RO5263397 (Thorn et al., 2014) were able to block cocaine relapse, further supporting the development of TAAR1-based pharmacotherapies in cocaine addiction. Data on potential interactions between METH and TAAR1 is much more limited. METH and cocaine have different actions on DAT with the former producing strong DA releasing effects. More importantly, while TAAR1 has no affinity for cocaine, activation of TAAR1 by METH results in inhibition of DA uptake, enhancement of DA efflux and DAT internalization (Xie and Miller, 2009). The present work was aimed at further characterizing the effects of TAAR1 activation on key METH-related behavioral and neurochemical changes. We tested the ability of the partial agonist, RO5203648, to modulate METH-induced locomotor activity and behavioral sensitization, METH self-administration and substitution, and METH-stimulated striatal DA efflux *in vitro* and *in vivo*.

## Materials and methods

### Subjects

Male Long Evans rats were sourced from the University of Canterbury, the University of Otago and the Italian Institute of Technology and were 8–10 weeks-old when experiments began. Rats were generated from outbred stocks, with rats from different institutions or suppliers not bearing any direct relationship. For the behavioral experiments, all animals were housed in a temperature and humidity controlled colony room with a 12-h light/dark cycle (lights off at 8 a.m.). Water and standard laboratory rat chow was given *ad libitum* at all times in all experiments except in the self-administration (S-A) experiment in which rats were given a maintenance diet (i.e., kept at 100% of their weight 7 days post-surgery) (Velazquez-Sanchez et al., 2011, 2013). To perform the [^3^H]dopamine uptake and efflux assays synaptosomes were prepared from brains of Wistar rats (F. Hoffmann-La Roche Ltd., Basel, Switzerland). Animal care and experimental protocols were conducted in compliance with the New Zealand Animal Welfare Act 1999, the Italian Ministry of Health (DL 116/92; DL 111/94-B) and European Community (86/609/EEC) directives, and the Swiss Federal and Basel Cantonal laws on animal research. All experiments were approved by the ethics committee affiliated to each institution. Power analysis was conducted as part of the applications for ethics approval to estimate the number of animals required per experiment, with α set at 0.05 and power at 90%. Data from all animals was included in the statistical analyses.

### Pharmacological agents

Methamphetamine hydrochloride was obtained from BDG Synthesis (Wellington, New Zealand) and dissolved in 0.9% physiological saline for intraperitoneal (i.p.) injection and intravenous S-A. RO5203648 (partial TAAR1 agonist) was synthesized at F. Hoffman-La Roche Ltd. (Switzerland) and dissolved in 10% dimethylsulfoxide and 0.9% physiological saline.

### Catheter implantation surgery

Rats used for the S-A experiment were anesthetized with Avertin (2,2,2-tribromoethanol, 12.5 mg/ml, in 2.5% tertiary amyl alcohol, 2 ml/100 g of body weight, i.p.). The analgesic carprofen was administered before surgery (5 mg/kg, i.p.). Catheters (O/D 0.63 mm, I/D 0.30 mm, Camcaths Cambridge, UK) were implanted into the right jugular vein, exiting dorsally between the scapulae. Analgesic and antiseptic cream was applied to the back and neck incision areas following suturing. To prevent infection rats were treated post surgically with daily injections of antibiotic (Cephalexin, 10 mg/kg, s.c.) for 7 days. Catheters were flushed with heparinised saline (0.1 ml, 70 IU/ml) before and after each S-A session.

### Behavioral procedures

#### Locomotor activity

Locomotor activity experiments were conducted in a set of four open field boxes made of black Perspex (50 × 40 × 35 cm). Locomotor activity was monitored and measured with a video tracking system and image analysis software (Viewpoint 2.5, Champagne au Mont D'Or, France) that provided automatic measures of traveled distance, trajectory and velocity of the subjects.

Six groups of rats (*n* = 5–6 per group) were habituated in the open field for 10 min for two consecutive days. During the test, rats were given a pretreatment of RO5203648 (0, 5, 10 mg/kg, i.p.) followed 15 min after by METH (0, 0.75 mg/kg, i.p.). 10 min after METH treatment, rats were placed into the open field and locomotor activity was measured for 3 h. Four rats were tested concurrently in four separate open fields. Locomotor activity was estimated as distance traveled and recorded in 20 min bins.

For the sensitization experiments, six groups of rats (*n* = 5–6 per group) were habituated to the open field during 30 min on two consecutive days. Because this experiment assessed the chronic effects of RO5203648 and METH, the doses selected were lower than that used in the 3-h locomotor experiment described above. During the 14 days sensitization period, rats received daily treatment of RO5203648 (0, 1.67, 5 mg/kg, i.p.) followed 10 min after by METH (0, 0.75 mg/kg, i.p.) and were allowed to freely explore the open field for 60 min. Four rats were tested concurrently in four separate open fields. Locomotor activity was recorded on alternate days. Treatments were administered in the home cages on the no-test days. Rats underwent withdrawal from all pharmacological treatments for three consecutive days before receiving a challenge with a low dose of METH (0.25 mg/kg, i.p.) to probe for sensitization. Locomotor activity was recorded for 60 min in the sensitization test.

#### S-A experiments

Eight operant conditioning chambers (Panlab, SL, Barcelona, Spain) controlled by software (Packwin software package) were used in the S-A experiments. Chambers were equipped with two response levers, an infusion pump, a house light and a stimulus light. Presses on the active lever resulted in activation of the infusion pump and delivery of METH or saline, illumination of a light stimulus for 5 s and initiation of a 20 s time-out. Presses on the inactive lever were recorded but had no programmed consequences. Each experimental chamber was enclosed in a light- and sound-attenuating box. The house light was on throughout training and test sessions.

After surgery, rats were randomly assigned to METH (*n* = 10) and saline groups (*n* = 6) and were trained to receive METH (0.05 mg/kg/infusion) or saline infusions under a fixed ratio (FR) 1 reinforcement schedule in daily 60 min sessions. METH intake tests began after the rats in the METH group met a criterion of consistency and stability (number infusions per session ≥15 for three consecutive days with less than 20% variability). Each rat completed three METH-taking tests on alternate days during which a pre-treatment of RO5203648 (0, 3, 10 mg/kg, i.p.) was administered 10 min before the self-administration session. The order in which treatments were administered was counterbalanced between subjects. After completion of all three tests rats were re-exposed to standard METH or saline self-administration sessions.

After the rats in the METH group returned to stable responding (number infusions per session ≥15), we tested self-administration of the TAAR1 partial agonist to examine its reinforcing properties. Each rat underwent four substitution tests in which varying doses of RO5203648 were given through intravenous infusions (0, 0.25, 0.5, and 1 mg/kg/infusion). Substitution tests were conducted on separate 60 min sessions, one for each dose, on alternate days. The order of the four tests was fully randomized. After all four substitution tests were carried out, rats completed a test in which the self-administered solution was replaced by a dose of METH three times weaker than the training dose (0.017 mg/kg/infusion; training dose was 0.05 mg/kg/infusion). This test was done in order to ascertain that the rats remained sensitive to variations in the reinforcing efficacy of the drug available for self-administration.

To test for potential nonspecific effects of RO5203648 on general motivation and performance rats were trained on saccharin (0.1%) self-administration. After two sessions of stable responding (≥15 reinforcements per session), each rat was tested three times, receiving a pretreatment of RO5203648 (0, 3, 10 mg/kg i.p.), administered in a counterbalanced fashion 10 min before the saccharin self-administration session started.

### Synaptosome [^3^H]DA uptake and efflux assays

Wistar rats were sacrificed at 12 weeks of age to collect brain samples. This strain was used because of the availability and reliability of standards on our extensive database and consistent results with a range of psychomotor stimulants. For each synaptosomal preparation we pooled 5 rat brains. Each experiment was performed in 3 technical replicates and 3–5 biological replicates. The striatum was dissected out and homogenized in 20 vol buffer A (0.32 M sucrose, 4 mM Hepes/NaOH, pH 7.4, 5 mM DTT, 1 mM EDTA and complete protease inhibitor) with a homogenizer using ceramic beads (Minilyser, Bertin technologies, France). The homogenate was centrifuged at 800 × g at 4°C for 10 min and the supernatant was kept. The pellet was resuspended in 5 vol buffer A and centrifuged again under the same conditions. Supernatants were pooled and centrifuged at 9000 × g at 4°C for 20 min. The pellet was resuspended in 10 vol buffer A and centrifuged again under the same conditions. The pellet was resuspended in 1 ml/brain recovery cell culture freezing media (LuBioScience, Lucerne, Switzerland). Protein concentration was determined and stored at −80°C until needed.

On the day of the uptake assay, striatal synaptosomes were washed in Krebs–Hepes buffer, incubated for 20 min at room temperature with various concentrations of METH (0.00003–30 μM) with or without co-application of 3 μM RO5203648, or with various concentrations of RO5203648 alone (0.00003–30 μM). The synaptosomes were then incubated with DA tracer for 10 min. After rigorous washing with PBS, scintillator (Perkin Elmer, Schwerzenbach, Switzerland) was added and the uptake of [^3^H]DA was detected with a liquid scintillation analyzer (TopLab, Switzerland).

For the efflux assay, synaptosomes were preloaded with [^3^H]DA and incubated in Krebs–Hepes buffer at 25°C for 20 min. After centrifuged at 4°C for 3 min (max speed), the synaptosomes were resuspended in buffer containing different concentrations of METH (0.3–10 μM) with or without co-application of 3 μM RO5203648. The synaptosomes were then incubated at 25°C for 45 min, washed again with ice cold buffer, and centrifuged, as previously, before scintillator was added and counted.

### *In vivo* microdialysis

*In vivo* microdialysis was performed in the right nucleus accumbens (NAc) of freely moving rats, using concentric microdialysis probes (membrane length 2 mm, cut-off 6000 Da; CMA-11, CMA/Microdialysis, Solna, Sweden). Animals were anesthetized with oxygen/isofluran mixture, and positioned in a stereotaxic apparatus. Stereotaxic coordinates for probes position were chosen according to the rat brain atlas: AP +1.7; L 1.4; DV −8.1 relative to bregma. Probes were implanted in the brain vertically through small drilled aperture in the skull and fixed with dental cement. The dialysis probes were perfused during implantation into the brain and for 1 h afterward with artificial cerebrospinal fluid (aCSF) (NaCl 147 mM, KCl 2.7 mM, CaCl_2_ 1.2 mM, MgCl_2_ 0.85 mM; CMA Microdialysis).

Approximately 24 h after surgery the dialysis probes were connected to a syringe pump and perfused with aCSF at 1.0 μl/min for 60 min (equilibration period) and then the perfusate was collected at a perfusion rate of 1.0 μl/min every 20 min for at least 60 min into collection tubes containing 2 μl of 1 M perchloric acid (basal levels). After this period animals were injected with vehicle, METH (0.75 mg/kg, i.p.) or RO5203648 (5 mg/kg, i.p.) or received double injections with RO5203648 and METH together (at the same doses) and the perfusate was collected at a perfusion rate of 1.0 μl/min every 20 min over a 3 h period. Quantification of DA in microdialysis samples was performed using HPLC with electrochemical detection (ALEXYS LC-EC system) equipped with a reverse-phase column (3 μm particles, ALB-215 C18, 1 × 150 mm, Antec Leyden BV, Netherlands) at a flow rate of 200 μl/min and electrochemically detected by a 0.7 mm glass carbon electrode (Antec; VT-03). The mobile phase contained 50 mM H_3_PO_4_, 50 mM citric acid, 8 mM KCl, 0.1 mM EDTA, 400 mg/l octanesulfonic acid sodium salt and 10% (vol/vol) methanol, pH 3.9. The sensitivity of the method permitted detection of ~3 fmol DA. Dialysate samples (11 μl) were injected into HPLC without any purification.

### Statistical analysis

Data were analyzed by analysis of variance (ANOVA) with repeated measures when a within-subjects design was in use. *Post-hoc* comparisons were conducted with the method of Newman–Keuls (N–K) using the sampling error from the overall ANOVA as denominator. Statistical significance was set at α = 0.05 for all experiments. All statistical analyses were performed using StatView 5.0 (SAS Institute, NC, USA).

## Results

### RO5203648 blocks METH-induced locomotor sensitization

**Figure 1:**
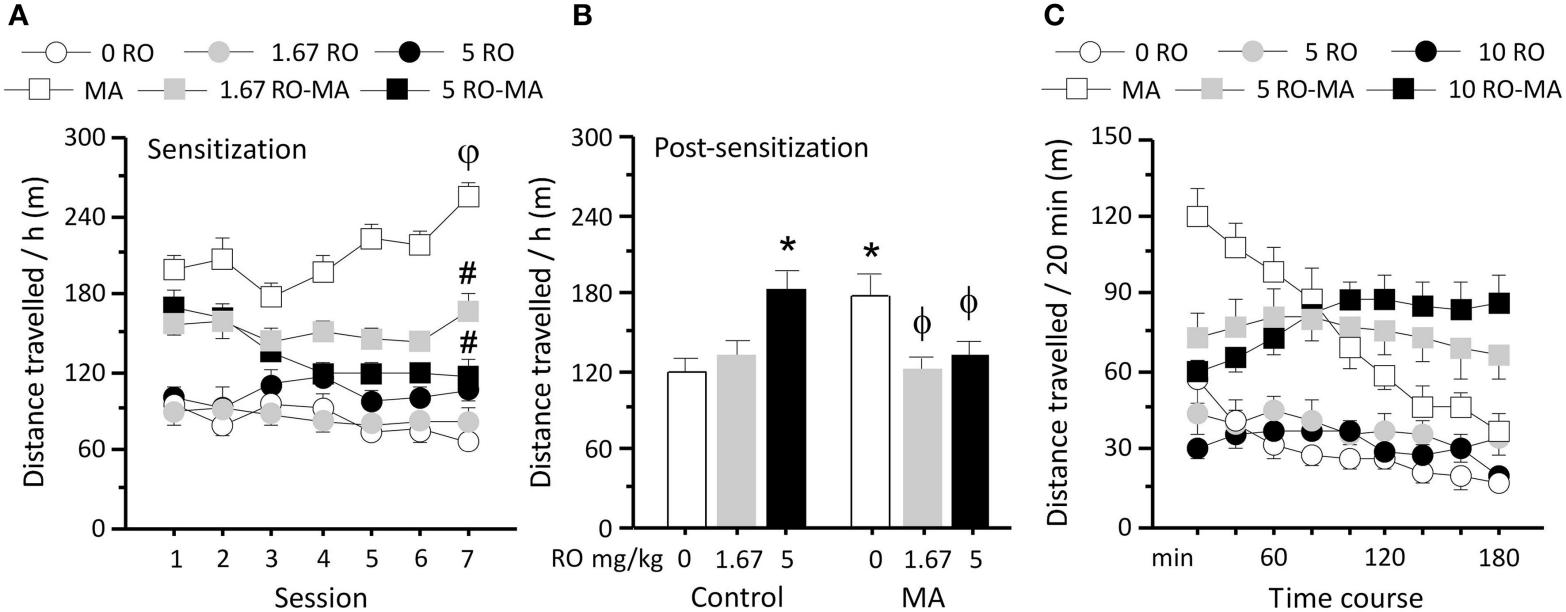
**Modulation of METH-induced locomotor activity and sensitization by TAAR1. (A)** Chronic exposure to methamphetamine gradually increased locomotor activity. Low and moderate doses of RO5203648 attenuated METH-induced locomotor activity and blocked the progressive increment induced by repeated exposure to METH. **(B)** Following withdrawal from chronic METH exposure, a challenge with a low dose of METH produced sensitized locomotor responsiveness. The long-term expression of locomotor sensitization was prevented in rats that had received concurrent treatment with RO5203648 (at either low or moderate doses) and METH. Treatment with the high dose of RO5203648 alone during the sensitization phase enhanced the subsequent long-term response to METH. **(C)** METH produced robust increases in locomotor activity compared with control treatment in a 3-h activity test. RO5203648 produced an early attenuation and a late potentiation of METH-stimulated locomotor activity. φ*p* < 0.01, different from acute METH (day 1); ^#^*p* < 0.01, different from chronic METH (day 7), ^*^p < 0.01 different from METH (after chronic control treatment), ϕ*p* < 0.01 different from METH (after chronic METH exposure). RO = RO5203648, MA = methamphetamine.

Rats were chronically treated with RO5203648 (0, 1.67, 5 mg/kg, i.p.), administered 15 min before METH (0, 0.75 mg/kg, i.p.), and locomotor activity was measured on alternate days. RO5203648 significantly attenuated locomotor sensitization induced by repeated METH treatment (Figure 1A). A repeated measure ANOVA yielded a significant main effect of drug treatment (*F*_5, 28_ = 26.68, *p* < 0.0001) and a significant interaction effect between the factors drug treatment, session (1 through to 7) and time (twelve 5 min time bins) (*F*_330, 1848_ = 1.19, *p* < 0.019). *Post-hoc* comparisons showed that METH significantly enhanced locomotor activity as treatments progressed, with activity of the 7th test being significantly higher compared with the 1st (*p* < 0.01, by N–K tests). The ability of RO5203648 to attenuate the effects of METH gradually increased across sessions. Both doses of RO5203648 were effective at reducing METH-induced locomotor activity (*p* < 0.05 for session 2 and 4, *p* < 0.01 for sessions 5 through to 7) and preventing METH sensitization during the acquisition phase (Figure 1A).

After 14 days sensitization treatment and 3-day withdrawal rats received METH (0.25 mg/kg, i.p.) and locomotor activity was evaluated. Previous repeated METH exposure in the sensitization phase led to a significantly heightened response to METH administration, which was significantly attenuated by RO5203648 (Figure 1B). ANOVA revealed a significant effect of treatment (*F*_5, 28_ = 3.27, *p* = 0.0189). *Post-hoc* comparisons indicated that rats with a history of chronic METH treatment showed significantly higher locomotor activity comparing to controls (*p* < 0.01, by N–K tests). This sensitized response was significantly reduced by both doses of RO5203648 (*p* < 0.01, by N–K tests). Moreover, RO5203648 treatment alone at the high dose, but not the low dose, produced significant cross-sensitization in the probe test as revealed by *post-hoc* comparisons (*p* < 0.01 comparing to control, by N–K tests).

### RO5203648 time-dependently modulates METH-induced locomotor activity

To study the interactions between RO5203648 and METH across time, locomotor activity induced by these treatments was examined for 3 h in an open field following single and combined administration. Rats received a pre-treatment of RO5203648 (0, 5 or 10 mg/kg, i.p.) 15 min before METH (0 or 0.75 mg/kg, i.p.). RO5203648 altered the effects of METH on locomotor activity in a time-dependent fashion, producing an early attenuation followed by a striking late potentiation. Locomotor activity was analyzed by repeated-measure ANOVA, which showed a significant effect of treatment (*F*_5, 29_ = 15.80, *p* < 0.0001) and time (*F*_8, 232_ = 15.94, *p* < 0.0001), as well as a significant interaction between these factors (*F*_40, 232_ = 8.35, *p* < 0.0001) (Figure 1C). METH produced high levels of locomotor activity in the first 20 min that decreased gradually over the 3 h test. RO5203648 significantly attenuated METH-induced hyperlocomotion during the first hour at both the low and the high doses (*p* < 0.01 for the 1st–3rd bins, by N–K tests). However, as the effect of METH treatment alone slowly subsided, RO5203648 potentiated METH's effects by maintaining locomotor activity at a moderately high levels, which were significantly higher than that produced by METH alone at both the low (*p* < 0.05 for the 6th bin, *p* < 0.01 for the 7–9th bins, by N–K tests) and the high dose of RO5203648 (*p* < 0.05 for the 5th bin, *p* < 0.01 for the 6–9th bins, by N–K tests).

### RO5203648 blocks METH, but not saccharin self-administration, and lacks reinforcing efficacy

**Figure 2:**
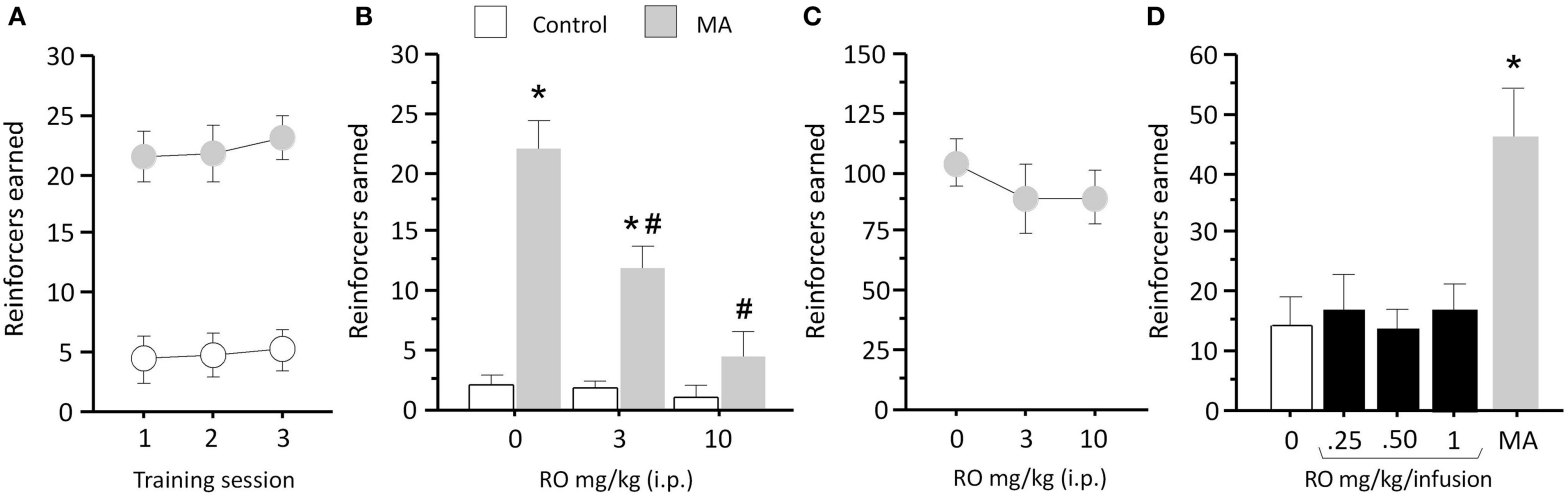
**RO5203648 decreases METH S-A and exhibits reduced abuse liability in a substitution test. (A)** Rats were trained on METH S-A until consistent performance was attained. **(B)** RO5203648 dose-dependently reduced METH S-A but did not affect sucrose S-A **(C)**. **(D)** In a substitution procedure, varying concentrations of RO5203648 did not sustain S-A behavior over and above control values, whereas a concentration of METH three times lower than that used during training generated strong responding. ^*^p < 0.01, different from control values; ^#^*p* < 0.01, different from METH. RO = RO5203648, MA = methamphetamine.

We then tested the ability of RO5203648 to attenuate METH S-A. Rats trained to self-administer METH under a FR1 schedule of reinforcement obtained a significantly higher number of infusions than rats responding for saline (*n* = 6) (*F*_1, 11_ = 34.45, *p* = 0.0001) (Figure 2A). Pre-treatment with RO5203648 (0, 3, 10 mg/kg, i.p.) significantly attenuated METH S-A (Figure 1B). ANOVA revealed a significant main effect of drug (METH vs. saline, *F*_1, 10_ = 23.39, *p* = 0.0007) and dose of RO5203648 (*F*_2, 20_ = 12.12, *p* = 0.0004), as well as a significant interaction between those factors (*F*_2, 20_ = 9.74, *p* = 0.0011). *Post-hoc* comparisons indicated significant effects of both doses of RO5203648 on the number of METH infusions obtained (*p* < 0.01, by N–K tests) (Figure 2B). The number of infusions obtained by the control group remained low and was not affected by RO5203648.

To control for potential motoric and motivational confounds, we tested the effects of RO5203648 on responding maintained by a natural reinforcer (saccharin). Pre-treatment with RO5203648 (0, 3, 10 mg/kg, i.p.) 10 min before the saccharin S-A session did not affect responding as revealed by a repeated measures ANOVA (*p* = 0.639) (Figure 2C).

To test the abuse potential of RO5203648, we substituted METH for RO5203648 in the S-A task. Rats received infusions of RO5203648 at different doses (0, 0.25, 0.5, 1.0 mg/kg/infusion, i.v.) on separate tests. In the final test rats worked for METH infusions (0.017 mg/kg/infusion). One-way ANOVA revealed a significant effect of the self-administered drug (*F*_4, 24_ = 11.67, *p* < 0.0001) (Figure 2D). *Post-hoc* comparisons showed that S-A of different doses of RO5203648 did not generate varying levels of responding over and above S-A of saline. However, S-A of METH maintained significantly elevated rates of responding compared with RO5203648 and saline infusions (*p* < 0.01, by N–K tests), with the number of METH infusions at the low dose being 2-fold greater than that obtained with the training dose (Figure 2B).

### RO5203648 has no effect on METH-induced DA release or inhibition of DA uptake in the striatum *in vitro*

**Figure 3:**
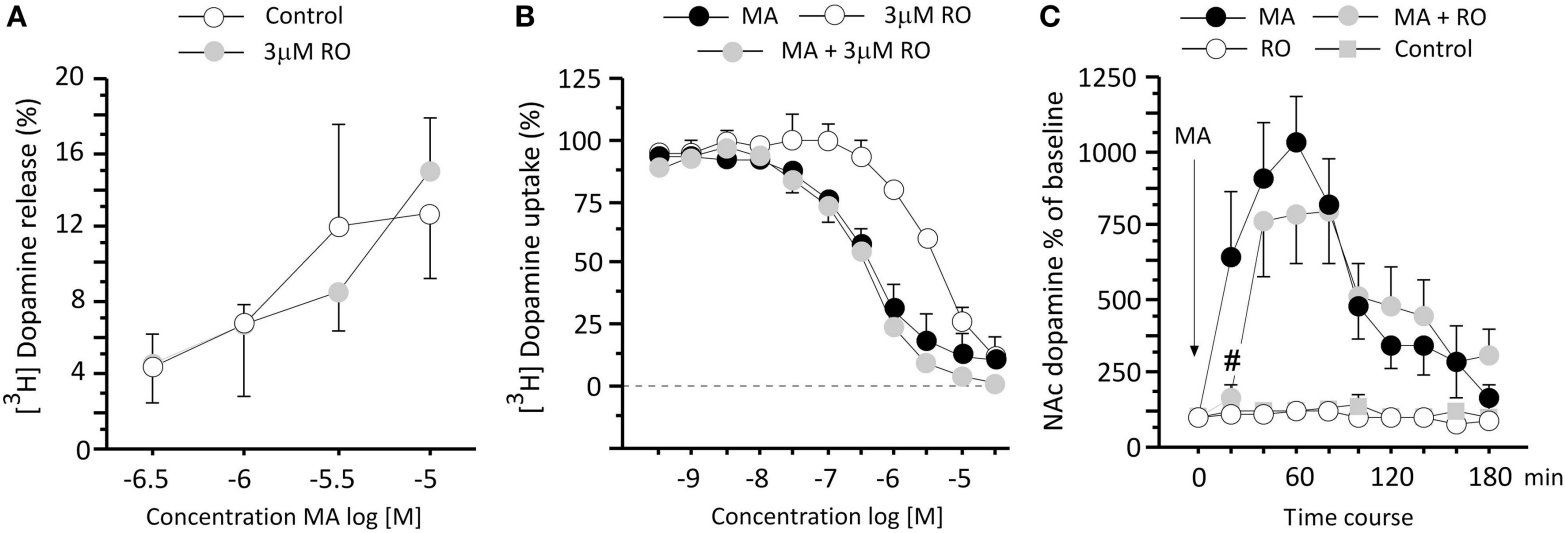
**RO5203648 transiently attenuated DA overflow in the NAc *in vivo* but not in striatal synaptosomes. (A)** In striatal synaptosomes the efflux of [^3^H]DA induced by varying concentrations of METH was not affected by co-application of RO5203648, as was the uptake of [^3^H]DA **(B)** In the 3-h long *in vivo* microdialysis experiment RO5203648 delayed the strong increases in extracellular DA accumulation induced by METH treatment in the NAc, producing a marked transient inhibition of the DA response **(C)** please note that a pure control cannot be included in the uptake experiments **(B)** because no efflux or release can be measured after vehicle treatment. ^#^*p* < 0.01, different from METH. RO = RO5203648, MA = methamphetamine.

In order to investigate the impact of TAAR1 partial agonism on METH-induced DA release and uptake, striatal synaptosomes were stimulated with various concentrations of METH to elevate synaptic levels of DA. METH-induced increases in DA transmission were not affected by the co-application of 3 μM RO5203648, as revealed by a two-way ANOVA which showed only a significant main effect of the METH treatment (*F*_3, 14_ = 4.56, *p* = 0.0199) (Figure 3A). In the uptake assay, METH induced a significant inhibition of DA uptake in a concentration-dependent manner. Similarly, uptake inhibition was not altered by the co-incubation with 3 μM RO5203648. RO5203648 alone also produced a weak DA uptake inhibition, but at a concentration (IC_50_ 3 μM) 10-folder higher than that produced by METH alone (IC_50_ 0.3 μM). A Two-Way ANOVA revealed a significant main effect of concentration (*F*_10, 44_ = 89.37, *p* < 0.0001) and a significant main effect of treatment (*F*_2, 44_ = 34.23, *p* < 0.0001), as well as a significant interaction between those factors (*F*_20, 44_ = 2.70, *p* = 0.0030) (Figure 3B).

### RO5203648 attenuates METH-induced changes in DA transmission in the NAc

To explore the mechanisms underlying the ability of RO5203648 to modulate METH-stimulated behaviors, we measured DA outflow in the NAc using *in vivo* microdialysis. Samples were collected for 3 h at 20 min intervals following treatment with METH, alone and in combination with RO5203648. A repeated measure ANOVA revealed a significant effect of treatment (*F*_3, 23_ = 20.16, *p* < 0.0001) and time (*F*_9, 207_ = 13.47, *p* < 0.0001), as well as a significant interaction between these factors (*F*_27, 207_ = 5.35, *p* < 0.0001). *Post-hoc* comparisons showed that METH produced a significant increase in DA level, which reached peak level at 60 min, followed by a decrease over time (*p* < 0.01 comparing to control, for the 1st–6th bins, by N–K tests). RO5203648 blocked the METH-induced increase in DA levels in the first 20 min after METH administration (*p* < 0.01 at 40 min, by N–K tests) (Figure 3C).

## Discussion

The present data provide extensive evidence of interactions between METH and the TAAR1 partial agonist, RO5203648, demonstrated in a range of behavioral paradigms and neurochemical experiments, including tests of locomotor activity, sensitization, self-administration, and *in vivo* microdialysis. Taking together, the current data showed a significant ability of TAAR1 to modulate the neurochemical and behavioral effects of METH.

METH is known for its ability to potently increase extracellular DA levels in the mesolimbic dopaminergic pathway, which largely underlies its motor-stimulating and rewarding effects. Following repeated exposure, METH produces long-lasting neuroadaptive changes in the mesolimbic DA system, characterized by a progressive augmentation of stimulant-induced DA efflux, which is thought to be a key neurochemical signature of METH behavioral sensitization (Yamada et al., 1988; Nakagawa et al., 2011). Our finding that repeated RO5203648 co-administered with METH blocked the development of METH sensitization suggests that TAAR1 is able to modulate the long-term neuroadaptations induced by chronic METH exposure. In line with these observations, it has been shown previously that mice lacking *taar1* were hypersensitive to the motor-stimulating effects of METH (Achat-Mendes et al., 2012), suggesting that TAAR1 is either constitutively active or tonically activated by ambient ligands to suppress the dopaminergic response to METH. In the current study, TAAR1 activation by RO5203648 may have exerted an inhibitory control over METH-evoked DA transmission, thereby preventing the induction of the dopaminergic hypersensitivity that underlies METH sensitization. Markedly complicating this picture, however, is the fact that METH itself is a potent full agonist at TAAR1, potentially leading to the initiation of phosphorylation events that down-regulate DAT function resulting in extracellular DA accumulation (Xie and Miller, 2009). Hence, alternatively or complementarily, RO5203648 may block METH-induced excessive DA transmission through the TAAR1-mediated pathway by directly competing with METH at TAAR1, thereby attenuating the progression of dopaminergic sensitization induced by repeated METH. Intriguingly, repeated RO5203648 alone, at moderate doses (5 mg/kg), cross-sensitized with METH, suggesting that long-term treatment with the partial agonist may have resulted in neuroadaptations in the mesolimbic DA system that are similar to those evoked by chronic METH treatment. Previous studies have shown that alterations of both D1 and D2 DA receptors play a key role in the development of stimulant sensitization (Shuto et al., 2006; McGinty et al., 2008). Although there is no evidence of direct interactions of TAAR1 with DA D1 receptors, previous data hints at a complex inter-relationship between TAAR1 and DA D2 receptors. Xie et al. (2008) demonstrated cross-attenuation of signaling between TAAR1 and DA D2 receptors, with their signaling having opposite effects on intracellular phosphorylation cascades and extracellular DA transmission (Xie et al., 2008). Additional findings revealed that *taar1* knockout mice displayed up-regulation of high-affinity striatal D2 receptors (Wolinsky et al., 2007) and reduced D2 receptor-mediated auto-inhibition (Leo et al., 2014). Thus, it is possible that intermittent partial activation of TAAR1 with RO5203648 alters the coordinated interactions between TAAR1 and DA D2 receptors or causes neuroadaptive changes in DA D2 receptors, leading to altered auto-inhibitory control over dopaminergic transmission. Alternatively or complementarily, repeated TAAR1 activation may lead to persistent changes in glutamatergic transmission. Enhanced glutamatergic transmission in the ventral tegmental area and nucleus accumbens plays a fundamental role in stimulant sensitization (Wolf, 1998; Vanderschuren and Kalivas, 2000). The full TAAR1 agonist, RO5166017, prevented the hyperactivity induced by NMDA receptor blockade (Revel et al., 2011), suggesting that TAAR1 activation may facilitate glutamate neurotransmission. Importantly, at the low dose (1.67 mg/kg) RO5203648 did not cross-sensitize with METH but maintained its ability to reduce METH sensitization. Therefore, it would appear that at low doses the partial TAAR1 agonist is able to block METH sensitization without inducing long-term METH-like neuroadaptations.

To further investigate the interactions between TAAR1 and METH, we conducted a 3-h locomotor activity test after acute treatment with RO5203648, alone and combined with METH. Early during the test RO5203648 produced a dose-dependent attenuation of METH-induced hyperactivity, which is consistent with our finding that partial TAAR1 activation attenuated METH-evoked DA release. Interestingly, RO5203648 potentiated the locomotor-enhancing effects of METH in the later phase of the locomotor activity test, as METH's effects began to decay. Our attempt to gain insight into the neurochemical mechanisms mediating this interaction was only partially successful. METH-induced DA accumulation in the NAc seemed to be altered by TAAR1 activation in biphasic fashion but the late potentiation effect on DA overflow did not reach statistical significance. This indicates that mechanisms other than net DA transmission at NAc synapses are likely to contribute to the complex regulation of METH-induced hyperactivity by TAAR1. Additionally, the later increase in locomotion is unlikely to be explained by a gradual temporal decay of RO5203648's effects, which could potentially allow the recovery of METH's effects over time, since this biphasic action of RO5203648 was also observed in a previous study in which RO5203648 alone produced an early inhibition followed by a later enhancement of food-maintained responding (Pei et al., 2014). In the experiment by Pei et al. rats treated with RO5203648 only (i.e., not combined with METH) responded for food over and above control rats under a progressive ratio schedule of reinforcement, with more responses accumulating long after treatment (3–6 h after), suggesting that RO5203648 remains psychoactive for several hours.

In the synaptosomal preparation RO5203648 did not affect METH-induced striatal DA release and DA uptake inhibition, suggesting that RO5203648 regulation of METH's behavioral effects is unlikely to depend on direct, local actions at the DAT. As previously indicated, the *in vivo* microdialysis data revealed transient but significant reduction in METH-induced DA overflow following RO5203648 treatment. The apparent discrepancy between the *in vitro* and *in vivo* experiments could be explained by the difference in concentrations used and the network effects of systemic TAAR1 activation. Indeed, TAAR1 seems to exert inhibitory control over the activity of DA-releasing neurons originating in the ventral tegmental area. The full TAAR1 agonist, RO5166017, decreased the firing rate of mouse midbrain DA neurons (Revel et al., 2011), whereas the effects of RO5203648 on DA transmission appeared to be dependent on ambient levels of endogenous agonists, including DA itself, given its partial agonist profile (Revel et al., 2012b). The synaptosomal assays were conducted with tissue that may have contained synaptic fractions from dorsal striatum, thus potentially adding variability to the assay. We also acknowledge the use of different strains of rat for the synaptosomal assays and microdialysis experiments. Thus, tissue-specific and strain-specific differences may have contributed to these inconsistent results.

The current findings also demonstrated that RO5203648 dose-dependently attenuated METH self-administration, which is likely to reflect a decrease in the reinforcing efficacy of METH, although we acknowledge that we only tested METH S-A under a FR1 schedule and at one dose (0.05 mg/kg/infusion). Our data are consistent with previous evidence indicating that the partial agonist RO5263397 reduced self-administration of METH at doses of METH that were both on the ascending and descending limbs of the dose-response curve (Jing et al., 2014). Further, we showed that the effect of RO5203648 on METH self-administration could not be attributed to general motivational or motor deficits because RO5203648 did not affect sucrose self-administration. Previous studies have shown that RO5203648 decreased cocaine self-administration and cocaine seeking at doses that did not impair food-maintained responding (Revel et al., 2012b; Pei et al., 2014). These observations are consistent with the finding that *taar1* deletion led to earlier acquisition and delayed extinction of place preference induced by METH exposure (Achat-Mendes et al., 2012), similarly suggesting an inhibitory control of TAAR1 over METH's rewarding properties. Importantly, our data also revealed that RO5203648 did not maintain significant levels of S-A when it was substituted for METH, which may be indicative of low abuse potential, a desirable feature for TAAR1-based pharmacotherapies to have. On the basis of these data, and considering that TAAR1 agonists have been shown to have clear therapeutic-like effects in models of cocaine addiction (Pei et al., 2014; Thorn et al., 2014), it may be tempting to speculate that such potential clinical application could be expanded to METH addiction. However, such generalization requires considerable caution given the complexities revealed by the present results, especially those that relate to long-term TAAR1 agonist treatment and behavioral sensitization.

In summary, the present data revealed novel complex interactions between the selective TAAR1 partial agonist, RO5203648, and METH. These findings further demonstrate the potential of TAAR1 to modulate stimulant-induced neurochemical and behavioral effects and provide additional support for the investigation of TAAR1 as a target for therapeutic intervention in addictive disorders.

### Conflict of interest statement

The authors declare that the research was conducted in the absence of any commercial or financial relationships that could be construed as a potential conflict of interest.
